# Clinical characteristics of “re-positive” discharged COVID-19 pneumonia patients in Wuhan, China

**DOI:** 10.1038/s41598-020-74284-6

**Published:** 2020-10-15

**Authors:** Shengyang He, Kefu Zhou, Mengyun Hu, Chun Liu, Lihua Xie, Shenghua Sun, Wenwu Sun, Liangkai Chen

**Affiliations:** 1 Respiratory and Critical Care Medicine Department, The Third Xiangya Hospital of Central South University, Changsha, China; 2 Intensive Care Unit, The Central Hospital of Wuhan, Tongji Medical College, Huazhong University of Science and Technology, Wuhan, China; 3 Department of Nutrition and Food Hygiene, Hubei Key Laboratory of Food Nutrition and Safety, Ministry of Education Key Lab of Environment and Health, School of Public Health, Tongji Medical College, Huazhong University of Science and Technology, Wuhan, China

**Keywords:** Medical research, Risk factors, Signs and symptoms

## Abstract

To analyze the clinical characteristics of re-positive discharged COVID-19 patients and find distinguishing markers. The demographic features, clinical symptoms, laboratory results, comorbidities, co-infections, treatments, illness severities and chest CT scan results of 267 patients were collected from 1st January to 15th February 2020. COVID-19 was diagnosed by RT-PCR. Clinical symptoms and nucleic acid test results were collected during the 14 days post-hospitalization quarantine. 30 out of 267 COVID-19 patients were detected re-positive during the post-hospitalization quarantine. Re-positive patients could not be distinguished by demographic features, clinical symptoms, laboratory results, comorbidities, co-infections, treatments, chest CT scan results or subsequent clinical symptoms. However, re-positive rate was found to be correlated to illness severity, according the Acute Physiology and Chronic Health Evaluation II (APACHE II) severity-of-disease classification system, and the confusion, urea, respiratory rate and blood pressure (CURB-65) score. Common clinical characteristics were not able to distinguish re-positive patients. However, severe and critical cases classified high according APACHE II and CURB-65 scores, were more likely to become re-positive after discharge.

## Introduction

The Corona Virus Disease 2019 (COVID-19), caused by severe acute respiratory syndrome coronavirus-2 (SARS-CoV-2), has had a worldwide impact since its first case in 2019. The genomic characteristics of SARS-CoV-2 was initially reported by Lu and colleagues, suggesting this coronavirus had enveloped RNA, resembling severe acute respiratory syndrome coronavirus (SARS-CoV) both structurally and homologically^1^. Until now, nearly 8 million people have been diagnosed with COVID-19, with more than 300 thousand deaths globally. Specifically, China has had more than 80 thousands confirmed cases with more than 3 thousands deaths, according to the World Health Organization (WHO) COVID-19 daily report dashboard (https://covid19.who.int/); with other countries including Italy, Korea, the United States, also reporting many confirmed cases, COVID has been officially declared a pandemic.

Previous studies have reported that some patients, after ‘recovering’ from the virus, could again test nucleic acid positive by RT-PCR^2,3^. However, the mechanisms behind this positive test result remain unclear. It is unknown whether these recovered patients may still be virus carriers. Considering the lack of detailed information regarding these patients and the lack longitudinal studies, the management of discharged COVID-19 patients is crucial.

In the present study, the clinical data of 267 confirmed COVID-19 patients who had been discharged from the Central Hospital of Wuhan, China, has been retrospectively analysed. Data showed that, 30 of 267 patients were again shown to be SARS-CoV-2 nucleic acid positive following 14 days quarantine. A further comparative analysis to explore the characteristics of these “re-positive” discharged patients was further performed. After reviewing their demographic characteristics, main symptoms, laboratory and radiology results, treatments and disease progression, no differences were found, suggesting this group of COVID-19 patients could be difficult to detect by using standard clinical data.

To formulate a more effective COVID-19 patients management strategy, in this investigation a novel method to asses and discriminate ‘re-positive patients’ infectivity from common COVID-9 patients is presented.

## Methods

### Study design and participants

Patients’ admission time ranged from January 1st to February 15th. COVID-19 diagnoses were made according to criteria from the *7th version of the guidelines on the Diagnosis and Treatment of COVID-19* issued by the National Health Commission of China. All raw clinical and laboratory results were collected from electronic medical records system of the Central Hospital of Wuhan, followed by a follow up visit up to 14 days (also known as the discharge quarantine) to test for a re-positive nucleic acid assay. All participants signed informed consent. The study was approved by the ethics committee of the Central Hospital of Wuhan and was performed in accordance with the principles of the Helsinki Declaration II.

### Data collection

All 267 patients enrolled in the study were from different COVID-19 units of the Central Hospital of Wuhan. All COVID-19 tests were performed by different departments of the Central Hospital of Wuhan. Computer Tomography (CT) scan evaluations were made by at least 2 specialists from the radiology department. The SARS-CoV-2 nucleic acid RT-PCR test quality control was performed by specialists from the clinical laboratory department. The clinical data included: the demographic descriptions, main symptoms, comorbidities, changes of laboratory results, main treatments, etc. For privacy reasons, the raw data of these patients are not presented.

### Clinical definition

COVID-19 diagnosis was performed by detecting SARS-CoV-2 RNA in nasopharyngeal swabs. The RNA detection kits were provided by Sansure Biotech (Changsha, China) and ZJ Bio-Tech (Shanghai, China), and used according manufacturer’s protocol by specialized laboratory personnel.

The severity of COVID-19 patients was defined according to the 7th version of the *Guidelines on the Diagnosis and Treatment of COVID-19*. Briefly, (1) mild type: mild clinical symptoms without any radiology findings; (2) general type: limited clinical symptoms: i.e. fever, cough and other common pneumonia related symptoms with radiological abnormality; (3) severe type: patients have any of the following: (a) respiratory distress, respiratory rate ≥ 30 per min; (b) oxygen saturation on room air at rest ≤ 93%; (c) partial pressure of oxygen in arterial blood/fraction of inspired oxygen ≤ 300 mmHg; (4) critical type: patients have any of the following: (a) respiratory failure occurs and mechanical ventilation is required; (b) shock occurs; (c) patients with other organ dysfunction needing intensive care unit monitoring treatment.

COVID-19 patients were considered discharged when they meet all the criteria from the 7th version of the *Guidelines on the Diagnosis and Treatment of COVID-19*. Briefly, (1) normal body temperature for more than 3 days; (2) significantly recovered respiratory symptoms; (3) lung imaging shows obvious absorption and recovery of acute exudative lesion; (4) negative results of the nucleic acid tests of respiratory pathogens for consecutive two times (sampling interval at least 1 day).

Definition of “re-positive”: when a confirmed COVID-19 patient is detected SARS-CoV-2 RNA positive during the 14 days post-discharge quarantine (random test timing).

### Laboratory confirmation and treatment

All laboratory results were double checked by at least 2 specialists from the clinical laboratory medicine department. Hospitalized patients were tested for SARS-CoV-2 RNA every 24–72 h before discharge. The brief indications for corticosteroid utility (intravenous injection) are described as follow: (1) respiratory distress, respiratory rate ≥ 30 per min; (2) deteriorations on radiology results after initial treatments; (3) oxygen saturation on room air at rest ≤ 93%.

### Statistics analysis

Continuous variables are presented as median (interquartile range, IQR) and categorical variables as n (%). Differences in clinical characteristics and laboratory findings between groups were compared using Mann–Whitney U test (continuous variables) and chi-squared test or Fisher’s exact test (categorical variables). The multivariate logistic regression has been made for data valid test. All analyses were performed using R software (The R Foundation, https://www.r-project.org, version 3.6.1). A two-sided significance level of 0.05 was used to evaluate statistical significance.

## Results

### Demographic features and clinical symptoms

267 COVID-19 patients admitted to the Central Hospital of Wuhan from January 2 to February 15, 2020 were enrolled in the present study. 30 out of 267 COVID-19 patients (Table 1) were detected ‘re-positive’ during the post-discharge quarantine. The demographic characteristics were found not to be associated with ‘re-positive’ rate. Common symptoms of hospitalized COVID-19 patients, including fever, muscle ache, fatigue, headache, cough, chest tightness, chest pain, and diarrhea were taken into consideration, and none of these clinical symptoms could account for the ‘re-positive’ outcome.

**Table 1:** Demographic and clinical symptoms of COVID-19 patients.

	All (n = 267)	Re-positive classification		*P* value
		No (n = 237)	Yes (n = 30)	
Age, years	57 (37–68)	57 (37–67)	66 (42–71)	0.07
Male	116 (43%)	99 (42%)	17 (57%)	0.12
**Chief complaint**				
Fever	212 (79%)	187 (79%)	25 (83%)	0.81
Muscle ache	59 (22%)	55 (23%)	4 (13%)	0.25
Fatigue	93 (35%)	87 (37%)	6 (20%)	0.07
Headache	18 (7%)	16 (7%)	2 (7%)	> 0.99
Cough	192 (72%)	167 (70%)	25 (83%)	0.19
Chest tightness	102 (38%)	11 (5%)	1 (3%)	0.54
Chest pain	12 (4%)	11 (5%)	1 (3%)	> 0.99
Diarrhoea	20 (7%)	19 (8%)	1 (3%)	0.71

### Comorbidities and co-infections

A large majority of patients (Table 2) had comorbidities such as hypertension (33%), diabetes (17%), chronic kidney disease (2%), lung diseases (7%) and tumor-related diseases (2%). Still, none of these co-morbidities were found to correlate with ‘re-positive’ patients. Furthermore, common co-infections (i.e. mycoplasma, chlamydia and other respiratory virus-Table 2) detected at admission or during hospitalization, were also found to have no correlation with ‘re-positive’ outcomes (Table 2).

**Table 2:** Comorbidities and co-infections of COVID-19 patients.

	All (n = 267)	Re-positive classification		*P* value
		No (n = 237)	Yes (n = 30)	
**Comorbidities**				
Chronic kidney disease	5 (2%)	4 (2%)	1 (3%)	0.45
Chronic pulmonary disease	20 (7%)	16 (7%)	4 (13%)	0.26
Hypertension	89 (33%)	77 (32%)	12 (40%)	0.41
Diabetes	45 (17%)	39 (16%)	6 (20%)	0.63
Cardiovascular disease	28 (10%)	22 (9%)	6 (20%)	0.07
Cerebrovascular disease	16 (6%)	14 (6%)	2 (7%)	0.7
Malignancy	7 (3%)	6 (3%)	1 (3%)	0.57
**Co-infection**				
Mycoplasma	17 (6%)	15 (6%)	2 (7%)	> 0.99
Chlamydia	9 (3%)	7 (3%)	1 (3%)	> 0.99
Influenza-A/B	0	0	0	NA

### Treatments and severities

According disease severity, different treatment plans were adopted, such as antibiotics including quinolone and cephalosporins and antivirus including ribavirin, oseltamivir, abidor and lopinavir/ritonavir. Methylprednisolone, intravenous gamma globulin (IVIG) and ventilation was also selectively utilized. No significance differences in treatments modalities, comparing with the re-positive patients (Table 3) were observed. However, severity of illness (as per classification described above) showed that the ‘re-positive’ patients tend to be severe, along with APACHE II and CURB-65 score, which are both indicators of severities. Similarly, the hospitalization length of stay and whole medical care costs results were consistent with APACHE II and CURB-65 (Table 3).

**Table 3:** Treatment and severities of COVID-19 patients.

	All (n = 267)	Re-positive classification		*P* value
		No (n = 237)	Yes (n = 30)	
**Treatment**				
Quinolone	179 (67%)	157 (66%)	22 (73%)	0.44
Cephalosporins	116 (43%)	100 (42%)	16 (53%)	0.25
Ribavirin	232 (87%)	206 (87%)	26 (87%)	> 0.99
Oseltamivir	53 (20%)	45 (19%)	8 (27%)	0.32
Abidor	91 (34%)	85 (36%)	6 (20%)	0.08
Lopinavir ritonavir tablets	27 (10%)	27 (11%)	0	0.053
Glucocorticoids	150 (56%)	132 (56%)	18 (60%)	0.65
Intravenous immunoglobulin	114 (43%)	104 (44%)	10 (33%)	0.27
Ventilation	25 (9%)	21 (9%)	4 (13%)	0.5
**Severity**				
Mild	0	0	0	NA
General	154 (58%)	143 (60%)	11 (37%)	**0.03**
Severe	88 (33%)	74 (31%)	14 (47%)	
Critical	25 (9%)	20 (8%)	5 (17%)	
ARDS	94 (35%)	79 (33%)	15 (50%)	0.07
CURB-65	0 (0–1)	0 (0–1)	1 (0–1)	**0.003**
SOFA	1 (0–2)	1 (0–2)	2 (1–3)	0.17
APACHE II	3 (1–4)	3 (1–4)	4 (2–5)	**0.02**
Days from onset to hospitalization, days	7 (5–10)	7 (5–10)	7 (2–12)	0.582
Duration of hospital stay, days	27 (20–36)	25 (19–34)	36 (30–44)	**< 0.001**
Hospitalization expenses, RMB	25,118 (15,018–39,915)	24,232 (14,771–39,196)	30,596 (21,537–51,332)	**0.01**

Bold font indicates *P* value < 0.05.

### CT scan outcomes

**Figure 1:**
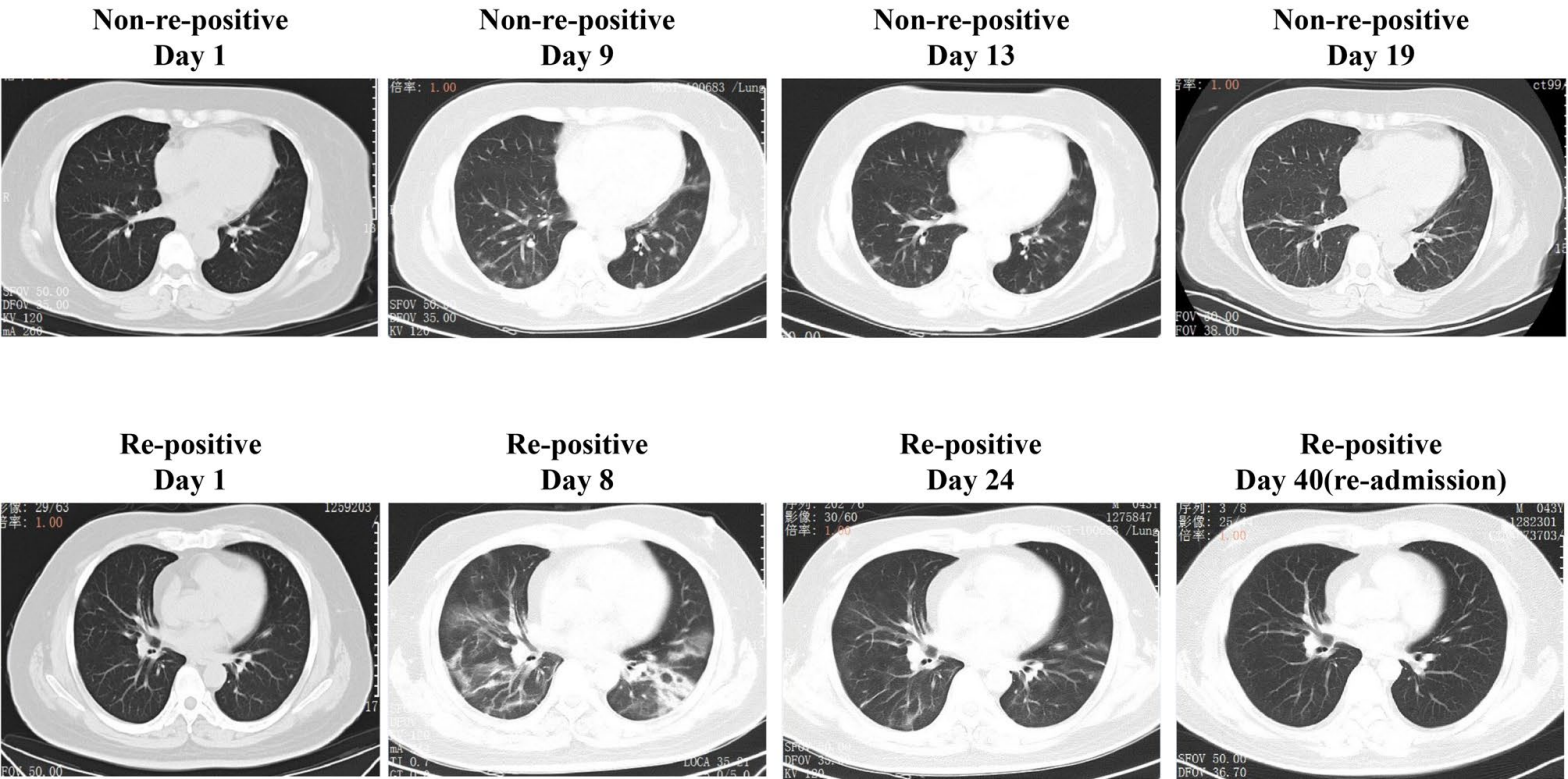
CT scan of representative patients in both non-re-positive patients and re-positive patients. Non-re-positive patients: Female, 61yo, few ground-glass opacities in bilateral lung field at day 1. Deterioration occurred subsequently (day9), and those lesions were absorbed in the following week (day 13 and day 19), accompanied by symptoms relief. Re-positive patient: male, 42yo, few ground-glass opacities in bilateral lung field at day 1. Deterioration occurred subsequently (day 8), and those lesions were absorbed in the following week (day 24), accompanied by symptoms relief. The lung lesions in CT scan were almost absorbed when found re-positive (day 40), without any recurrence of clinical symptoms.

To simplify the CT scan evaluation, all the enrolled patients were divided into three group. Group 1: lesions present in 0–30% of the bilateral lung field; and Group 2: 31–60%; 3: more than 61%, respectively. CT scan outcomes were found statistically insignificant (Table 4). The representative CT scan developments of both re-positive and non-re-positive patients are shown in Fig. 1.

**Table 4:** CT of discharged COVID-19 patients.

	All (n = 267)	Re-positive classification		*P* value
		No (n = 237)	Yes (n = 30)	
**CT severity**				
0	127 (48%)	111 (47%)	16 (53%)	0.61
1	75 (28%)	66 (28%)	9 (30%)	
2	65 (24%)	60 (25%)	5 (17%)

### Laboratory results

Routine blood tests, other blood biochemistry and blood gas analysis results at day 1, 3, 7 and day14, are presented in Table 5. No statistically differences were identified in ‘re-positive’ patients.

**Table 5:** Laboratory results of COVID-19 patients.

	All (n = 267)	Re-positive classification		*P* value
		No (n = 237)	Yes (n = 30)	
**White blood cell count, × 109/L**				
D1	4.9 (3.8–6.4)	4.9 (3.8–6.4)	4.8 (4.2–7.3)	0.461
D3	5.8 (4.3–7.6)	5.8 (4.3–7.5)	5.6 (4.2–8.9)	0.851
D7	6.0 (4.4–8.1)	6.0 (4.5–8.0)	6.3 (4.2–8.9)	0.834
D14	6.1 (4.6–8.0)	6.3 (4.7–8.0)	5.3 (4.4–8.0)	0.341
**Lymphocyte count, × 109/L**				
D1	1 (0.7–1.4)	1 (0.7–1.4)	1 (0.8–1.3)	0.974
D3	1 (0.7–1.3)	1 (0.7–1.3)	0.9 (0.7–1.2)	0.438
D7	1.2 (0.8–1.6)	1.2 (0.8–1.6)	1.1 (0.8–1.5)	0.479
D14	1.3 (1.0–1.7)	1.3 (1.0–1.7)	1 (0.9–1.5)	0.024
**Neutrophil count, × 109/L**				
D1	3.2 (2.3–4.7)	3.2 (2.3–4.6)	3.7 (2.5–5.4)	0.209
D3	4 (2.8–5.7)	4 (2.8–5.6)	3.9 (2.7–7.1)	0.857
D7	4 (2.7–6.2)	4 (2.8–6.1)	3.9 (2.5–6.5)	0.975
D14	4 (2.9–5.8)	4.1 (3.1–5.9)	3.8 (2.6–5.6)	0.571
**Platelet, × 109/L**				
D1	189 (142–243)	186 (140–239)	216 (176–260)	0.02
D3	212 (166–282)	214 (166–284)	191 (157–260)	0.556
D7	220 (179–282)	217 (179–280)	228 (156–290)	0.919
D14	229 (176–291)	230 (176–292)	216 (176–274)	0.746
**Hemoglobin**				
D1	126 (116–138)	126 (116–138)	127 (118–136)	0.717
D3	123 (113–134)	123 (114–134)	125 (109–136)	0.969
D7	118 (106–130)	118 (105–129)	120 (110–133)	0.327
D14	110 (96–123)	109 (95–123)	114 (102–126)	0.343
**C-reactive protein, mg/dL**				
D1	1.4 (0.3–4.3)	1.3 (0.3–4.3)	1.7 (0.5–4.2)	0.806
D3	1.1 (0.3–3.2)	1.1 (0.3–3.0)	1.1 (0.3–4.3)	0.69
D7	0.4 (0.1–2.0)	0.4 (0.1–1.8)	0.4 (0.2–3.0)	0.443
D14	0.2 (0.1–0.8)	0.2 (0.1–0.7)	0.5 (0.1–3.0)	0.109
**PCT**				
D1	0.05 (0.04–0.08)	0.05 (0.04–0.08)	0.05 (0.04–0.09)	0.456
D3	0.06 (0.04–0.11)	0.06 (0.04–0.10)	0.05 (0.04–0.15)	0.76
D7	0.06 (0.04–0.08)	0.05 (0.04–0.07)	0.07 (0.05–0.09)	0.044
D14	0.05 (0.04–0.07)	0.05 (0.04–0.07)	0.06 (0.05–0.10)	0.019
**Blood urea nitrogen, mmol/L**				
D1	4.2 (3.3–5.2)	4.1 (3.3–5.1)	4.3 (3.3–6.9)	0.457
D3	4.7 (3.8–6.0)	4.7 (3.7–6.0)	4.8 (4.2–6.7)	0.335
D7	4.6 (3.6–5.9)	4.6 (3.6–5.9)	4.7 (3.7–6.4)	0.689
D14	4.3 (3.6–5.4)	4.3 (3.7–5.4)	4.5 (3.6–5.8)	0.572
**Creatinine, μmol/L**				
D1	65 (52–78)	65 (52–78)	66 (51–85)	0.803
D3	63 (50–76)	62 (49–73)	69 (52–82)	0.119
D7	64 (51–78)	62 (50–77)	75 (58–82)	0.076
D14	64 (49–76)	61 (49–75)	76 (53–84)	0.097
**Alanine aminotransferase, U/L**				
D1	19 (13–32)	19 (13–32)	18 (12–29)	0.315
D3	25 (15–46)	24 (16–47)	28 (14–45)	0.979
D7	24 (15–45)	23 (15–44)	37 (16–70)	0.115
D14	27 (15–50)	28 (14–48)	26 (17–64)	0.784
D1	2.8 (2.4–3.3)	2.8 (2.4–3.3)	2.9 (2.5–3.4)	0.317
D3	2.9 (2.4–3.4)	2.9 (2.4–3.4)	2.9 (2.5–3.5)	0.529
D7	2.5 (2.2–2.9)	2.5 (2.2–2.9)	2.6 (2.3–3.3)	0.279
D14	2.5 (2.2–2.9)	2.4 (2.2–2.8)	3.0 (2.3–3.4)	0.014
**D-dimer, μg/L**				
D1	0.59 (0.29–1.51)	0.59 (0.29–1.45)	0.58 (0.28–1.75)	0.74
D3	1.33 (0.63–4.23)	1.33 (0.65–4.18)	1.31 (0.56–3.80)	0.956
D7	1.02 (0.47–2.60)	1.01 (0.44–2.74)	1.03 (0.64–2.33)	0.84
D14	0.97 (0.41–1.92)	0.97 (0.40–1.90)	0.98 (0.53–2.65)	0.698
**Lactate dehydrogenase, U/L**				
D1	188 (152–235)	187 (151–236)	193 (157–231)	0.894
D3	204 (156–260)	203 (156–259)	212 (188–265)	0.601
D7	181 (151–233)	178 (150–223)	215 (152–263)	0.262
D14	172 (141–203)	168 (141–202)	186 (159–204)	0.304
**Creatine kinase, U/L**				
D1	77 (47–128)	77 (47–135)	76 (45–102)	0.595
D3	44 (30–70)	44 (30–67)	40 (27–152)	0.78
D7	36 (24–58)	36 (24–58)	32 (19–51)	0.759
D14	38 (26–57)	37 (25–51)	54 (31–64)	0.073
**Creatine kinase–MB, U/L**				
D1	7 (6–11)	7 (6–12)	7 (5–11)	0.588
D3	8 (6–12)	8 (6–12)	8 (7–11)	0.767
D7	7 (5–10)	7 (5–10)	8 (5–12)	0.855
D14	6 (4–8)	6 (4–8)	6 (5–9)	0.24
PaO_2_:FiO_2_, mmHg	343 (263–500)	350 (273–502)	316 (252–455)	0.23
Lactate, mmol/L	1.2 (0.8–1.9)	1.2 (0.8–1.9)	1.3 (1.0–1.9)	0.466

### Follow-up survey

To further evaluate the recovery of COVID-19 patients, a phone follow-up visit was set up with each patient, focusing on the incidence of clinical symptoms (Table 6). Results showed that many patients still had symptoms, including coughing (21%), phlegm (13%), palpitate (34%), chest tightness (25%), paracenesthesia (12%) and fatigue (48%). Additionally, 9 out of 30 ‘re-positive’ patients spent their quarantine at home with family, and no other family member have been reported to have been infected so far. A following study is underway to assess further outcomes of these “re-positive” patients.

**Table 6:** Resettlement location and follow-up visits of COVID-19 patients.

	All (n = 267)	Re-positive classification		*P* value
		No (n = 237)	Yes (n = 30)	
Home		66	9	
Isolation point		171	21	
Cough	55 (21%)	49 (21%)	6 (20%)	0.93
Phlegm	34 (13%)	28 (12%)	6 (20%)	0.21
Chest tightness	66 (25%)	56 (24%)	10 (33%)	0.25
Fatigue	128 (48%)	115 (49%)	13 (43%)	0.59
Palpitation	90 (34%)	81 (34%)	9 (30%)	0.65

### Multivariate logistic regression of potential predictors for “re-positive”

To further testify the predictors for “re-positive” cases, a multivariate logistic regression was conducted (Table S1). The OR were found greater than 1 for all predictors when using univariate test. Moreover, when adjusted by age (> = 65 yo), cardiovascular comorbidities, severities and the utility of glucocorticoids, OR values were decreased but still greater than 1, indicating potential predicting abilities.

## Discussion

Since the outbreak of the COVID-19 crisis in late 2019, millions of people have been diagnosed all over the world. Fortunately, the majority of hospitalized COVID-19 patients have been successfully discharged. However, many studies have reported that discharged patients could again be tested viral nucleic acid positive^2–5^, arising the possibility of a potential re-infection.

Results in this study showed that ‘re-positive’ patients do not display any distinguishing clinical markers, except illness severity, making the existence of such group of patients questionable.

Researchers questioned the low sensitivity of many viral RNA detection kit currently in use, that could also be affected by many other factors (e.g. quality control of the kits, quality and sample delivery method, etc.)^6^. Xiao and colleagues found this re-positive phenomenon could be due to false-negative of RT-PCR, since they observe that a certain number of COVID-19 patients had a prolonged viral RNA conversion time^7^. Yuan and colleagues retrospectively studied 25 re-positive COVID-19 patients in Shenzhen, China and suggested the results of viral nucleic acid by RT-PCR were variable, even if patients showed two negative results for respiratory pathogens nucleic acid tests before discharge^8^. This study, like ours, suggests that the re-positive phenomenon could be a technical bias rather than actual patient group.

Besides the possibility of false-negative results from RT-PCR, sample selection and collection could also lead to the ‘re-positive’ detection and efficient virus load could be key to have positive RT-PCR results. It has been shown that SARS-CoV-2 binds to ACE2 receptor which are mainly located in lower respiratory tract rather than upper^9^. Consequently, for instance, collected samples from a nasopharyngeal swab might have less virus load compared to other sample collected from the lower respiratory tract samples (e.g. alveolar lavage fluid), leading to an unreliable RT-PCR result.

Furthermore, even though samples are properly collected and analysed resulting in positive results, it could potentially not prove that patients are infective, as only those who can transmit live virus are defined as infective patients^10^.

Some researchers argued that the use of corticosteroid may have potential risks, as it could suppress our immune functions, decreasing the ability of viral clearance^11^. Theoretically, this could be a reasonable hypothesis, accounting for the occurrence of re-positive cases, however, in the present study, the use of corticosteroid did not increase the number of ‘re-positive’ patients, consistent with previous work by Lan^2^.

Lan and colleagues found those ‘re-positive’ patients to be younger, with shorter hospitalization time and shorter seroconversion. However, in the present study, the opposite was found. Specifically, ‘re-positive’ patients showed to be severe cases, with higher APACHE II and CURB-65 score, and longer hospitalization time.

Another issue to consider is the many differences between COVID-19 patients from China, Europe and America, especially with sequalae. For instance, researchers reported patients from Europe and America with more olfactory and gustatory complaints^12,13^, while Chinese patients had less^14^. One hypothesis could be related to the different virus strains present in different countries^15^, leading to different clinical characteristics and even sequalae. However, there are currently no study comparing “re-positive” rate in different regions worldwide.

Some limitations of the present study merit consideration. Firstly, no COVID-19 mild cases patients were enrolled in this study due to different local medical care policies. Specifically, Wuhan was the first city with COVID-19 outbreak, with the largest cases patients in the country. To increase medical care efficiency, many Fangcang shelter hospitals were created for mild COVID-19 cases^16^. Therefore, the Central Hospital of Wuhan, as a large general hospital, mainly dealt with patients ranging from moderate to critical. Additionally, at the beginning of COVID-9 outbreak in Wuhan, every large general hospital was overloaded, which may have resulted in an imperfect quality control of sample collection and delivery. Consequently, patients enrolled in this study are different to previous published result by Lan, which may lead to bias. Moreover, in Lan’s study, the severity of their enrolled patients were different from ours, as most of the COVID-19 patient in their study are general cases which may also cause bias. Additionally, a novel pathogen as SARS-CoV-2 is, it is currently not certain that what, the virus itself or the excessive immune reaction, account for the severity of patients. Therefore, it remains possible that severe and critical patients may have higher viral loads and longer clearance time. More research is necessary.

Furthermore, our is a single-center, retrospective study with limited number of participants, therefore, more prospective clinical research is needed.

Another important issues in our study is that viral load quantification was not conducted due to lack of skilled laboratory personnel at the beginning of this pandemic. Yu and colleagues reported that the quantitative virus load detection would give rise to the diagnosis sensitivity and accuracy, especially in cases with low virus load^17^.

Some researchers hypothesized that the re-positive COVID-19 cases could be the virus re-infection^18^. Immunologically speaking, after the acute infection of the SARS-CoV-2, the human body should generate specific neutralizing antibodies against the virus for at least 7 days^19^; furthermore a recent animal experiment in rhesus macaque indicated re-infection phenomenon did not happen^20^. These result are in line with other studies on severe acute respiratory syndrome (SARS)^21^ and middle east respiratory syndrome (MERS)^22^.

In conclusion, in the present study, 30 ‘re-positive’ COVID-19 patients were compared to 237 non-‘re-positive’ patients, showing no significant differences between these two groups based on clinical characteristics, but correlated to illness severity. No evidence indicates ‘re-positive’ patients were still infective, and those who have had close contacts with ‘re-positive’ patients were currently safe, but follow up studies are in progress.

Since understanding of the mechanisms of SARS-CoV-2 infection is still lacking, a careful discharge protocol should be applied (e.g. negative results of the nucleic acid tests of respiratory pathogens for 3 consecutive times), and post-discharge quarantine should be strictly observed, especially for severe and critical COVID-19 patients.

## Supplementary information

Supplementary Information.
