# Supplementary material for: Clinical characteristics of “re-positive” discharged COVID-19 pneumonia patients in Wuhan, China
Source: Sci Rep. 2020 Oct 15;10:17365. doi: 10.1038/s41598-020-74284-6 (PMC7562721; doi:10.1038/s41598-020-74284-6)
Supplement: Supplementary file 1 — Supplementary Information. [file 41598_2020_74284_MOESM1_ESM.docx]

**Clinical Characteristics Analysis of the “Re-positive” Discharged COVID-19 Pneumonia Patients in Wuhan, China**

Shengyang He, MD, PhD ^1#^, Kefu Zhou, MD^1#^, Mengyun Hu, BS^1^, Chun Liu, MD^1^, Lihua Xie, MD, PhD^1^, Shenghua Sun, MD, PhD^1^, Wenwu Sun, MD^2^*, Liangkai Chen, PhD^3^

1. Respiratory and Critical Care Medicine Department, The Third Xiangya Hospital of Central South University, Changsha, China.
2. Intensive Care Unit, The Central Hospital of Wuhan, Tongji Medical College, Huazhong University of Science and Technology, Wuhan, China.
3. Department of Nutrition and Food Hygiene, Hubei Key Laboratory of Food Nutrition and Safety, Ministry of Education Key Lab of Environment and Health, School of Public Health, Tongji Medical College, Huazhong University of Science and Technology, Wuhan, China.

* Corresponding authors: Wenwu Sun, Email: [sunwenwuzxyy@163.com](mailto:sunwenwuzxyy@163.com); Liangkai Chen, Email: [clk@hust.edu.cn](mailto:clk@hust.edu.cn)

# Contributed equally.

**Table S1. Multivariate logistic regression of potential predictors for “re-positive”**

|  | Univariate | P-value | Multivariate | P-value |
| --- | --- | --- | --- | --- |
| Age, y |  |  |  |  |
| <65 | 1 | - | 1 | - |
| >=65 | 2.42 (1.12, 5.22) | 0.024 | 1.90 (0.80, 4.51) | 0.15 |
| Male vs. female | 1.82 (0.85, 3.92) | 0.12 | 1.51 (0.68, 3.38) | 0.31 |
| Hypertension | 1.39 (0.64, 3.02) | 0.41 |  |  |
| CVD | 2.44 (0.90, 6.62) | 0.079 | 1.49 (0.48, 4.64) | 0.5 |
| COPD | 2.12 (0.66, 6.84) | 0.21 |  |  |
| Diabetes | 1.27 (0.49, 3.31) | 0.63 |  |  |
| Stroke | 1.14 (0.25, 5.27) | 0.87 |  |  |
| CKD | 2.01 (0.22, 18.59) | 0.54 |  |  |
| Cancer | 1.33 (0.15, 11.42) | 0.79 |  |  |
| Sevirity |  |  |  |  |
| General | 1 | - | 1 | - |
| Severe | 2.46 (1.06, 5.69) | 0.035 | 1.90 (0.78, 4.64) | 0.16 |
| Critically ill | 3.25 (1.02, 10.33) | 0.046 | 2.50 (0.73, 8.64) | 0.15 |
| Glucocorticoids | 1.19 (0.55, 2.59) | 0.65 | 1.04 (0.45, 2.39) | 0.92 |
